# Development of a Framework for Echocardiographic Image Quality Assessment and Its Application in CRT-D/ICD Patients

**DOI:** 10.3390/jcm15031055

**Published:** 2026-01-28

**Authors:** Wojciech Nazar, Damian Kaufmann, Elżbieta Wabich, Justyna Rohun, Ludmiła Daniłowicz-Szymanowicz

**Affiliations:** 1 Laboratory of Experimental and Translational Allergology, Department of Allergology, Faculty of Medicine, Medical University of Gdańsk, Smoluchowskiego 17, 80-214 Gdańsk, Poland; 2 1st Department of Cardiology, University Clinical Centre, Smoluchowskiego 17, 80-214 Gdańsk, Poland; 3 Department of Cardiology and Electrotherapy, Faculty of Medicine, Medical University of Gdańsk, Smoluchowskiego 17, 80-214 Gdańsk, Poland; damian.kaufmann@gumed.edu.pl (D.K.); elzbieta.wabich@gumed.edu.pl (E.W.); justyna.rohun@gumed.edu.pl (J.R.); ludmila.danilowicz-szymanowicz@gumed.edu.pl (L.D.-S.)

**Keywords:** transthoracic echocardiography, heart failure, image quality, cardiac resynchronisation therapy, implantable cardioverter defibrillator

## Abstract

**Background/Objectives:** Low image quality reduces diagnostic accuracy. We wanted to develop a framework for assessing transthoracic echocardiography (TTE) image quality in apical 2-, 3-, and 4-chamber views, and to use this framework to characterise segment-level visualisation patterns in patients with heart failure (HF). **Methods:** In this cross-sectional study, 268 TTE examinations from 230 patients qualified for ICD/CRT implantation in primary prevention of sudden cardiac death were analysed. Patient demographic, electrocardiographic, echocardiographic, and clinical characteristics were collected, and apical 2-, 3-, and 4-chamber views were extracted for image quality evaluation. Mean scores for each segment were calculated. The proportion of well-visualised segments per view was also evaluated. Risk factors for poor image quality were assessed. **Results:** We internally assessed the reliability of the framework (intra-class correlation coefficient > 0.9). The anterior and anterolateral walls consistently demonstrated the poorest quality, and the inferior segments the best. Clear inner-edge-to-outer-edge delineation of ≥5 segmental borders was achieved in only 30% of studies, while ≥5 endocardial border segments were visualised in 65% of cases. Reduced quality was frequently observed in patients with higher BMI and BSA, presence of HF risk factors (diabetes, prior myocardial infarction, and atrial fibrillation), and heart abnormalities (increased left ventricular end-diastolic value and hypokinesis). **Conclusions:** The prevalence of imaging challenges in TTE examinations performed in patients qualified for CRT-D/ICD implantation is high. These findings underscore the need for thorough training of echocardiographers and for sustained attention to technical details affecting image quality to achieve consistently high-quality images in routine practice.

## 1. Introduction

Transthoracic echocardiography (TTE) remains a fundamental imaging modality in the diagnostic evaluation of cardiovascular diseases [1,2,3,4,5]. The quality of echocardiographic images is of paramount importance for accurate quantitative assessment of the heart [1,2,3,4,5,6]. In particular, myocardial wall deformation analysis using speckle-tracking echocardiography (STE), along with all derived parameters—including global longitudinal strain (GLS), left ventricular work indices, and mechanical dispersion—is particularly sensitive to artefacts and poor image quality. It is well established that poor image quality significantly increases the risk of measurement errors, particularly for parameters that require a sharply defined left ventricular myocardial border, including deformation indices [1,2,3,4,5]. The more an echocardiographic parameter relies on artificial intelligence-based (AI-based) image analysis and advanced mathematical computations, the more critical the underlying image quality becomes [1,2,3,4,5,6]. As the clinical importance of STE-derived measurements continues to grow, ensuring their reliable assessment is critically important [3,7,8,9].

Existing approaches to guideline-based echocardiogram quality assessment and image acquisition standardisation focus on functional reproducibility rather than image quality itself (Table 1) [3,10]. Strain-based measures and vendor-specific tools are affected by inter-vendor variability, while CMR, although a reference for functional assessment, is not designed to evaluate echocardiographic image quality in routine practice [3,10,11,12]. AI-derived metrics and automated quantification methods are promising but remain heterogeneous, dependent on training data, and limited in clinical implementation [13,14,15,16]. Vendor-neutral platforms primarily address post-processing variability and do not account for acquisition-related limitations [17].

In our study, we focused on patients with heart failure (HF) eligible for an implantable cardioverter defibrillator (ICD) or a cardiac resynchronisation therapy defibrillator (CRT-D) in primary prevention of sudden cardiac death. Currently, qualification for these procedures follows the European Society of Cardiology (ESC) guidelines and is based only on the left ventricular ejection fraction (LVEF) and (for CRT-D candidates) QRS duration [8,9]. Within this population, we applied a proposed framework based on fine-grained border assessment, consisting of structured manual evaluation of endocardial and epicardial border delineation at the segmental level, combined with global qualitative image quality parameters. This approach offers high anatomical granularity, explicit assessment of both inner and outer myocardial borders, and vendor-independent evaluation of local and global determinants of image quality. Although the framework is time-consuming and requires trained readers, it provides a robust foundation for future automation using artificial intelligence-based tools, for example, total image score prediction.

Common pitfalls associated with suboptimal visualisation of the left ventricle include apical foreshortening and inadequate delineation of the myocardial–endocardial interface in specific LV segments [3,7,18]. These issues could be exacerbated in patients with reduced LVEF—a group that commonly exhibits increased LV volumes and anatomical or physiological conditions that hinder image acquisition by increasing heart-to-probe distance, such as obesity or hyperinflated lungs due to chronic obstructive pulmonary disease (COPD) related to long-term smoking [19].

Therefore, maintaining high-quality echocardiographic imaging seems crucial in clinical practice, including for patients qualified for ICD/CRT-D implantation [3,7]. To date, however, the issue of image quality in routine echocardiographic examinations in this specific patient population has not been systematically investigated.

### Aim

This study aimed to develop and evaluate a fine-grained system for assessing TTE image quality in apical views, and to use this framework to characterise segment-level visualisation patterns in patients with HF undergoing evaluation for ICD/CRT implantation. We further sought to identify clinical and echocardiographic factors associated with reduced image quality and to quantify the prevalence of common imaging challenges.

## 2. Methods and Materials

This retrospective, cross-sectional study was conducted in accordance with the Strengthening the Reporting of Observational Studies in Epidemiology (STROBE) guidelines [20].

### 2.1. Inclusion Criteria

In this study, we included patients aged ≥18 years who were admitted between 2018 and 2022 to the Department of Cardiology and Electrotherapy, Medical University of Gdańsk, for ICD/CRT-D implantation in accordance with the 2021 European Society of Cardiology (ESC) guidelines in the primary prevention of sudden cardiac death [8,9].

All patients had symptomatic HF with LVEF ≤ 35% despite at least 3 months of optimised guideline-directed medical therapy (GDMT) [8,9].

Patients were required to have complete clinical and demographic data, including medical history, a 12-lead electrocardiogram, and relevant laboratory parameters, available for review at the time of device qualification. In addition, at least one comprehensive transthoracic echocardiographic examination prior to device implantation (or, for ICD recipients, within one week before or after implantation) was required for inclusion.

Patients who did not meet any of the above inclusion criteria were excluded from the study.

### 2.2. Exclusion Criteria

- Lack of echocardiographic data in the system.
- Unstable or inadequate echocardiographic imaging, including significant probe tilting or translation, insufficient frame rate, and absence of one or more of the required apical views.
- Any clinical exclusion for ICD/CRT implantation (need for correction of severe structural cardiac abnormalities, incomplete coronary revascularisation, etc.).

### 2.3. Echocardiographic Image Extraction

From each eligible study, best-quality end-diastolic to end-systolic cine loops depicting the apical two-chamber (A2C), three-chamber (A3C), and four-chamber (A4C) views were selected.

From each cine loop, the end-diastolic frame was extracted for further analysis. This frame was deliberately selected as it poses the most significant challenge for image optimisation, representing the largest ventricular volume and dimensions, and thus the widest myocardial contour. Despite being the most technically demanding frame, it holds critical importance for both conventional echocardiographic quantification and advanced AI-based strain and geometric analyses.

Patients with dyskinetic wall motion abnormalities were excluded, as in such cases the end-diastolic frame may not correspond to the true maximal ventricular volume.

### 2.4. Final Study Cohort

**Figure 1:**
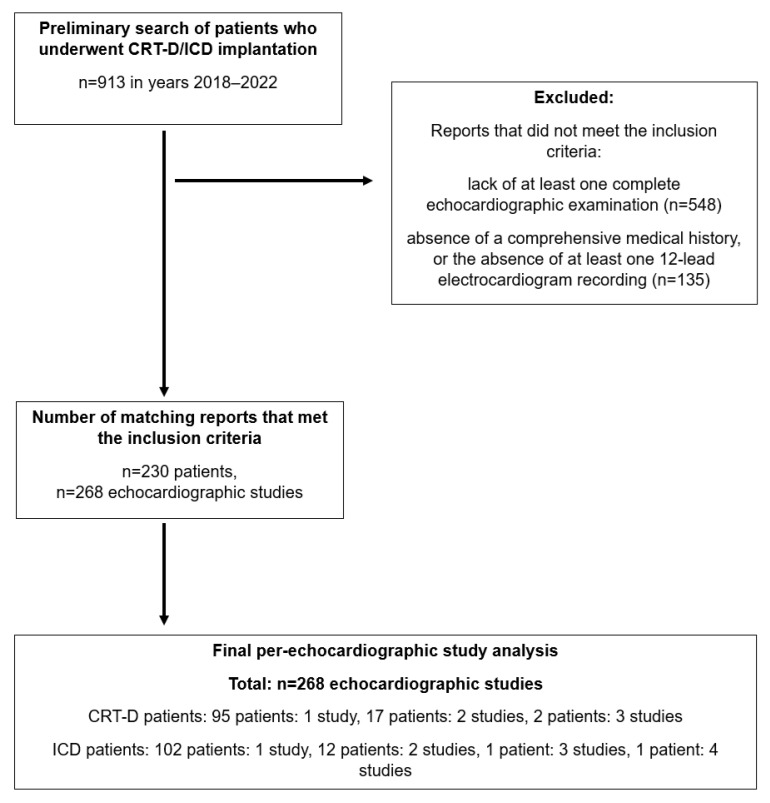
Study design.

An initial screening identified 913 patients who underwent ICD or CRT-D implantation between 2018 and 2022 (Figure 1). Following application of the predefined eligibility criteria, 230 patients were included in the final analysis—116 with ICD indications and 114 with CRT-D indications.

Multiple echocardiographic examinations from the same patient were included if they met all inclusion criteria, were performed by different echocardiographers and/or were separated by a minimum interval of one month.

Images were acquired using General Electric (GE) Healthcare VIVID E9 and E95 ultrasound systems. The measurements were performed using ViewPoint 6/EchoPAC software v206 from GE (Chicago, IL, USA).

Bi-plane echocardiographic measurements were obtained from the apical 2- and 4-chamber views, and the resulting values were averaged.

### 2.5. Primary Outcome-Image Quality Assessment Framework

For each extracted end-diastolic frame, image quality and anatomical visibility were systematically evaluated. To ensure consistent and anatomically detailed assessment, the left ventricular myocardium was divided into 18 standard segments according to the recommendations of the ESC/ASE [3,7]. Each frame was independently assessed by a board-certified cardiologist with at least five years of experience in echocardiography.

### 2.6. Scoring System and Weighting

The visual scoring system was expert-derived and developed to standardise the assessment of image interpretability. Weighting of the individual components was discussed within a group of experienced echocardiographers and cardiologists, and a consensus-based system was subsequently developed. Border delineation was considered the most critical determinant of image quality and, therefore, was assigned the greatest relative weight, while additional image quality parameters were included to capture complementary aspects of overall image usability. In our opinion, the selected components best differentiate images of poor versus good quality, which is supported by the ability of the score to identify images spanning a wide range of quality within the study cohort.

### 2.7. Segmental Border Assessment

Each myocardial segment was defined by two borders—endocardial (inner) and epicardial (outer)—and both were evaluated using a custom semi-quantitative 4-point scale:

1. Well-defined border—sharply demarcated, excellent image quality (3 points).
2. Moderately defined border—mildly blurred (2 points).
3. Indistinct border—very poorly visualised, but within the imaging sector (1 point).
4. Non-diagnostic—border outside the imaging field (0 points).

The sum of points for all segmental borders was expressed as the total border score (maximally 42 points per view), representing the quantitative measure of anatomical delineation within the frame. Additionally, if both the inner and outer borders of a segment scored 2 or 3 points, these were summed and defined as the total well-visible border score (+1 for each inner-edge-to-outer-edge well-visible border). Moreover, the inner border was assessed independently, and all segments with an endocardial border score of 2 or 3 were aggregated to derive the total well-visible endocardium score (+1 for each well-visible endocardium).

### 2.8. Global Image Quality Scoring

In addition to segmental analysis, an overall visual assessment was performed, evaluating the following qualitative parameters:

- First-sight image quality (1–5 points; 5 = excellent).
- Presence of artifacts (4 = none, 2 = moderate, 1 = numerous).
- Gain adjustment (4 = optimal, 2 = partially optimal, 1 = incorrect).
- Imaging axis alignment (3 = correct, 1 = incorrect).
- Apical foreshortening (3 = absent, 1 = present).

The sum of these components was calculated as the total image quality score (maximally 61 per view).

### 2.9. Image Quality Internal Reproducibility Assessment

To evaluate intra- and inter-observer reproducibility, 40 randomly selected patients (120 images) were independently re-analysed.

### 2.10. Statistical Analysis

Categorical variables were summarised as absolute counts and percentages. Group differences were analysed using contingency tables and the Chi-square test with Yates’ continuity correction for continuity, where appropriate.

Continuous variables, including quantitative image quality scores, were expressed as medians with 95% confidence intervals (95% CI). For data visualisation, mean scores were also calculated. The Shapiro–Wilk test was applied to assess the normality of data distribution. For normally distributed variables, parametric tests like the *t*-test and analysis of variance (ANOVA) were used. For non-normally distributed variables, nonparametric tests were applied—the Mann–Whitney U test and the Kruskal–Wallis test.

During the internal assessment, agreement between measurements was assessed using the intra-class correlation coefficient (ICC), coefficient of variation (CV), and Bland–Altman analysis to determine the mean bias and limits of agreement.

Linear relationships between continuous variables were evaluated using the Pearson correlation coefficient (PCC). The clinical strength of agreement for correlation and reproducibility metrics (PCC and ICC) was interpreted as follows: excellent, ICC ≥ 0.80; good, 0.60 ≤ ICC < 0.80; moderate, 0.40 ≤ ICC < 0.60; and poor, ICC < 0.40 [21].

For machine learning modelling, Extreme Gradient Boosting (XGBoost) classification was used to identify the top 10 predictors of echocardiographic image quality and quantify their relative contributions (feature importance). For classification, images were divided into two classes based on the median endpoint score to find features predictive of poor image quality: 0 (good image quality) and 1 (poor image quality). Cross-validation was not performed, as the objective was exploratory—to identify and rank the determinants of image quality—rather than to optimise predictive accuracy.

All analyses were conducted using Python 3.10 (Python Software Foundation) with NumPy 1.23.5, Pandas 2.1.3, XGBoost 2.0.1, and Scikit-learn 1.2.1 libraries. A two-sided *p*-value *p* < 0.05 was considered statistically significant.

## 3. Results

### 3.1. Demographic Characteristics

The study included 230 subjects: 116 patients with ICD indication and 114 patients with CRT-D indication (overall median age 66 years; 79% male, Table 2). The ICD and CRT-D groups were comparable, with no statistically significant differences in comorbidities or pharmacological therapy (*p* > 0.05), except for the median age of the patients (65 and 68 years, respectively; *p* = 0.013). NYHA class II was observed in 72% of patients, while NYHA class III was present in 28%.

In ECGs performed before device implantation (*n* = 268; Table 3), sinus rhythm was documented in 94% of all examinations. Median QRS duration was 165 ms in CRT-D and 112 ms in ICD (*p* < 0.001). LBBB was observed in 84% of CRT-D and 8% of ICD studies (*p* < 0.001), whereas RBBB occurred in 14% and 3% (*p* < 0.001), and IVCD in 4% and 18% (*p* < 0.001), respectively.

### 3.2. Echocardiographic Parameters

Bi-plane TTE measurements were largely similar between CRT-D and ICD cohorts (Table 3). In the analysed studies, median LVEDV was 197 mL, median LVESV was 145 mL, and median LVSV was 53 mL, with a median LVEF of 27%. Moderate tricuspid and mitral regurgitation were observed in 14% and 48% of studies, respectively. Atrial fibrillation (AF) during echocardiography occurred in about 6% of cases.

### 3.3. Echocardiogram Image Quality Analysis

For all studies, the median total image quality score per TTE study was 119.0 (out of a maximum of 183 points), whereas the median total border score across all views was 78 (out of a maximum of 126 points; Table 3). The median total first-sight image quality score for all views was 9 per study.

**Figure 2:**
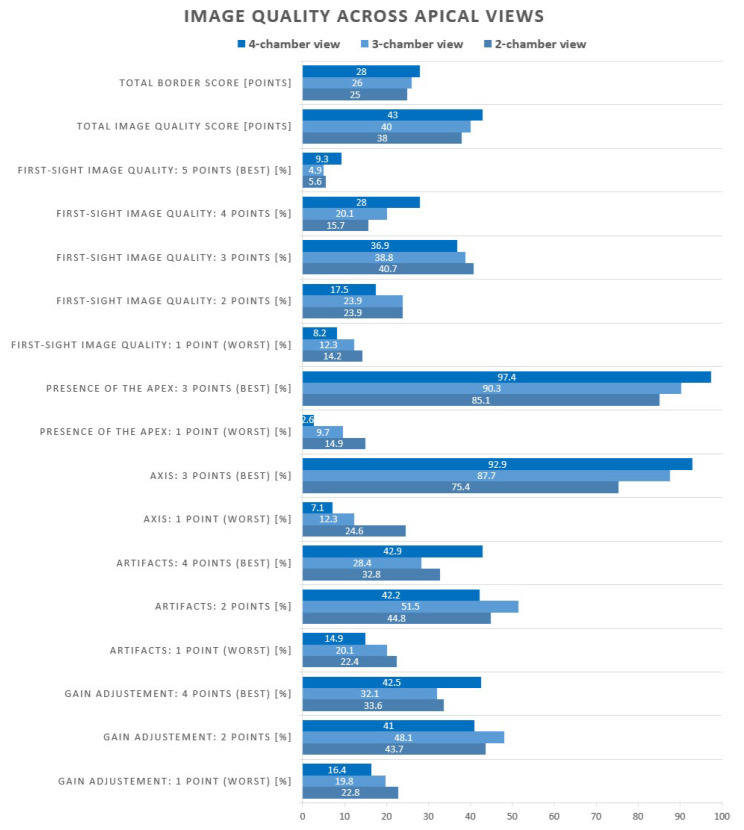
Comparison of image quality across apical views.

The total image quality score and total border points differed significantly across apical views (Figure 2; Supplementary Material S2). The median total image quality score increased progressively from 38 in the A2C view to 40 in the A3C view and 43 in the A4C view (*p* < 0.001). Similarly, the median total border points were 25 for A2C, 26 for A3C, and 28 for A4C (*p* < 0.001).

Proper visualisation of the cardiac axis was achieved in 75% of A2C acquisitions, whereas the A3C and A4C views demonstrated higher rates of adequate alignment, at 88% and 93%, respectively (*p* < 0.001, Figure 2). The apex was clearly depicted in 85% of A2C recordings, improving to 90% in A3C and 97% in A4C images (*p* < 0.001). Numerous artefacts occurred most frequently in the A2C view, noted in 22% of studies, compared with 20% in the A3C and 15% in the A4C view (*p* = 0.004). Suboptimal gain settings were most common in the A2C view (23%), with slightly lower rates in the A3C (20%) and A4C (16%) views (*p* = 0.060).

**Figure 3:**
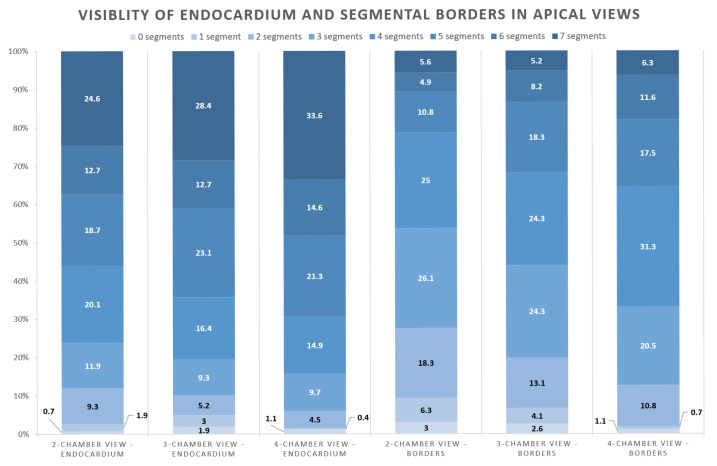
Visibility of endocardium and inner-edge-to-outer-edge segmental borders in apical views.

Assessment of border visibility showed that at least five inner-edge-to-outer-edge defined borders were present in 21% of A2C studies, compared with 32% in A3C and 35% in A4C examinations (*p* = 0.001; Figure 3; Supplementary Material S2). Scoring of endocardial border visibility indicated that five or more segments were adequately visualised in 56% of A2C, 64% of A3C, and 69% of A4C views (*p* = 0.095).

**Figure 4:**
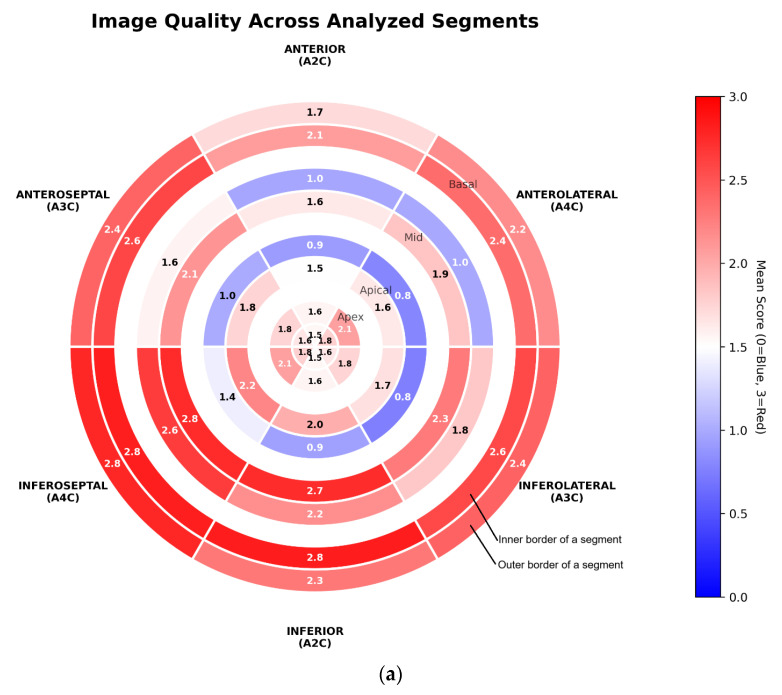

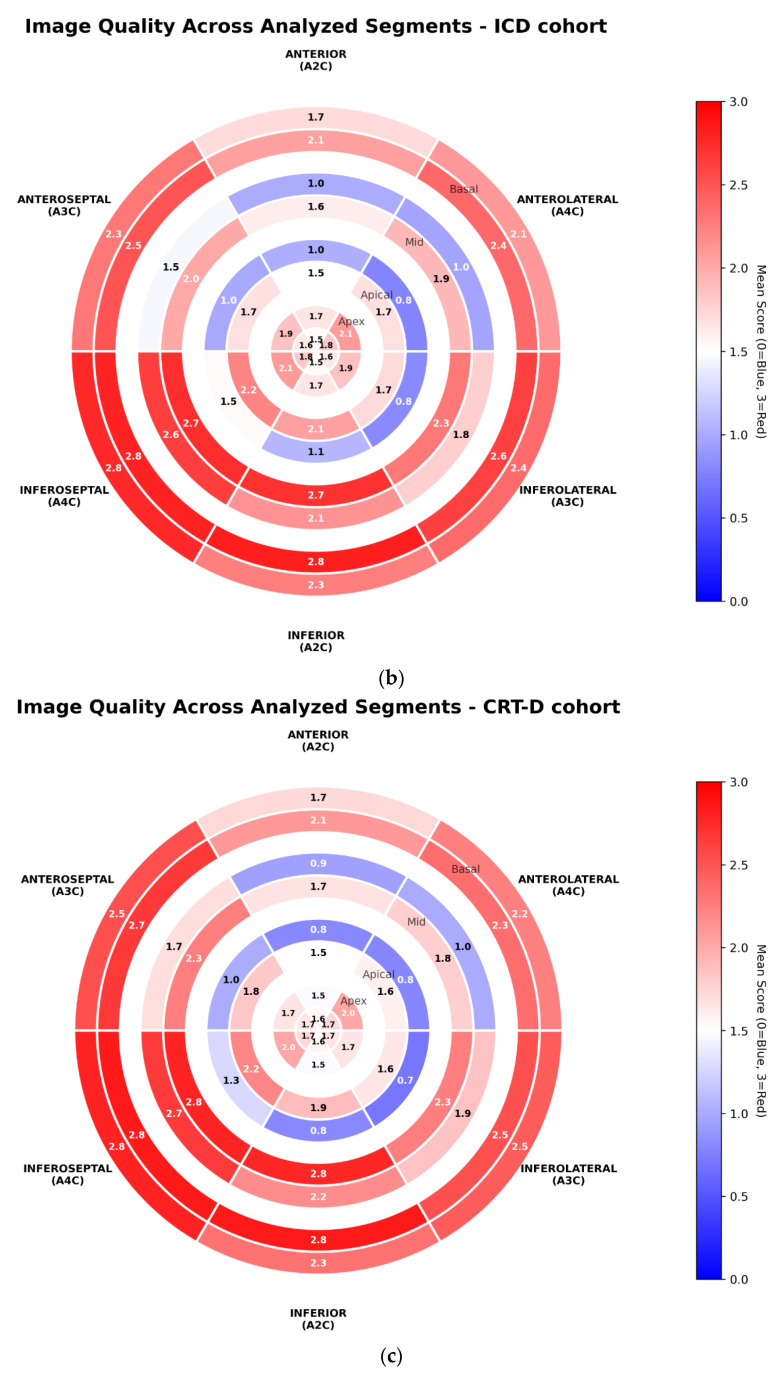
(**a**–**c**) Mean border-segment scores across analysed segments ((**a**): whole cohort; (**b**): ICD cohort; (**c**): CRT-D cohort).

Overall, the anterior wall demonstrates the poorest visualisation, as indicated by the lowest mean scores across border–segment pairs (Figure 4). The anterolateral wall in the A4C view shows the second-lowest visualisation quality. Conversely, the inferoseptal, inferior, and inferolateral segments show the best overall visualisation. Additionally, across all views and segments—particularly within the mid and apical regions—the outer border consistently exhibits lower mean scores than the inner endocardial border.

Subgroup analyses performed separately in the ICD and CRT-D cohorts yielded results highly consistent with each other and closely aligned with the findings in the overall study population (Supplementary Materials S1 and S2).

### 3.4. Pearson Correlation Coefficients

The total image quality score demonstrated a strong positive correlation with the total well-visible border score (r = 0.864; Supplementary Material S2), the total well-visible endocardium score (r = 0.882), the total border score (r = 0.964), and the total first-sight image quality score (r = 0.830). The total border score was also strongly associated with the total well-visible endocardium score (r = 0.850) and the total well-visible border score (r = 0.909).

### 3.5. Machine Learning Analysis

Across all evaluated models, no single parameter demonstrated a uniformly dominant influence on echocardiographic image quality (Supplementary Material S2). Nevertheless, among the most predictive variables for poor image quality, several factors consistently corresponded to reduced cardiac function, increased cardiac chamber dimensions, and several patient-related characteristics known to complicate ultrasound window acquisition.

For the total image quality score, the most influential factors encompassed clinical (history of AF, hypercholesterolemia, diabetes type 2, BMI, and male sex) and echocardiographic (hypokinesis of segment 15 and LVEDV) parameters. For the total border score, the principal predictors included hypercholesterolemia, hypokinesis of segment 8, BMI, age, patient height and weight, body surface area, and hypokinesis of segment 12. For the total well-visible endocardium score, the strongest predictors were clinical data such as coronary artery disease, previous MI, BMI, patient weight, and a history of PCI or CABG. Finally, for the total well-visible border score, some of the most relevant predictors included hypokinesis of segment 17, heart failure hospitalisation within the previous 12 months, patient weight, age, AF during the echocardiographic examination, BMI, or increased LVIDs.

### 3.6. Internal Reliability Assessment of the Image Quality Questionnaire

The intra- and inter-observer variability analyses demonstrated consistently high and clinically acceptable reproducibility across all evaluated image quality metrics. In every comparison, the ICC exceeded 0.90, indicating excellent reliability (Table 4). As anticipated, intra-observer measurements showed lower mean bias values (0.7 for the total border score and 0.5 for the total image quality score) than inter-observer assessments (0.8 and 1.3, respectively). In addition, the 95% confidence interval limits of agreement were narrower for intra-observer comparisons, further confirming superior consistency within readers. The mean absolute percentage error for intra-observer evaluations was 4.9% for the total border score and 4.0% for the total image quality score. In contrast, inter-observer error was higher at 8.8% and 7.9% for the respective parameters.

## 4. Discussion

(1) In this cross-sectional study of 268 TTE examinations from 230 ICD/CRT-D candidates, we introduce a reliable, fine-grained scoring framework for apical view image quality assessment.
(2) The anterior and anterolateral walls showed the poorest visualisation, whereas inferior segments had the highest quality; clear inner-edge-to-outer-edge delineation of ≥5 borders occurred in only 30% of studies, while ≥5 endocardial border segments were visible in 65% of cases.
(3) Machine learning analysis revealed no single determinant of image quality, though poorer visualisation was linked to reduced cardiac function, chamber enlargement, and patient factors known to impair acoustic windows.

Currently, no guideline-based framework exists for TTE image quality assessment, and objective standardisation remains challenging. Early studies relied on operator experience, using 2- or 3-category qualitative scales (poor/good or poor/medium/good) or simple criteria such as myocardial or landmark visibility [6,22,23,24]. Multi-level scales (e.g., 1–10) have also been proposed, though largely qualitative [25,26], as well as multidomain scoring systems such as that by Li et al. [27], which remain subjective—e.g., “average quality” is defined as “some contours are missing” without specifying acceptable limits or border prioritisation. A more objective approach by Huang et al. validated TTE-derived measurements (e.g., strain) against cardiac MRI, labelling images with the largest bias relative to MRI as poor-quality [2]. While robust, this method is costly, resource-intensive, and not feasible for routine practice. Additional challenges include the weighting of score components (border definition vs. global image quality) and the limited comparability across studies using different systems and cohorts. Inter-vendor variability in strain parameters, such as LVGLS, has decreased [22,28,29], yet commercial algorithms remain closed source; some, e.g., General Electric Healthcare, can exclude poorly tracked segments without disclosing the criteria or image features used [30].

Current ESC and ASE guidelines emphasise that the accuracy of strain analysis depends strongly on image quality. Significant sources of error include apical foreshortening, inadequate visualisation of myocardium or endocardial borders, inappropriate myocardial ROI size, acquisition during ectopic beats, and misidentification of end-diastole or end-systole, among others [3,10]. Consequently, poor image quality directly compromises diagnostic accuracy and clinical decision-making.

Guideline-based echocardiographic quality assessments prioritise functional reproducibility over intrinsic image quality (Table 1) [3,10]. Strain metrics and vendor-specific tools are hindered by inter-vendor variability, while cardiac MRI, though a functional reference standard, is not suited for routine image quality evaluation [3,10,11,12]. AI-driven metrics show promise but remain data-dependent and inconsistent in clinical practice [13,14,15,16]. Vendor-neutral platforms mitigate post-processing variability but do not address acquisition-related constraints [17].

In contrast, our proposed framework offers a structured, anatomy-based assessment of image quality that is independent of vendor and advanced post-processing, combining fine-grained manual evaluation of endocardial and epicardial borders for all segments—including the apex—with global qualitative parameters such as gain, artefact burden, and axis alignment. Although time-consuming, this approach provides a detailed and transparent characterisation of acquisition-related limitations, is well-suited for future AI-based automation, and represents a level of segmental granularity not previously reported in clinical studies. Our method balances objectivity, detailed border analysis, and practicality. Internal reliability assessment shows strong correlations between endocardial/segmental visibility and both total border and image quality scores (r > 0.8; Supplementary Material S2). Intra- and inter-observer reproducibility is excellent, with ICC > 0.9 for both scores and mean absolute errors of about 3–4% (intra-rater) and 6–8% (inter-rater; Table 4).

In our cohort, the anterior wall showed the poorest visualisation, as reflected in the lowest mean scores across border–segment pairs (Figure 4). The anterolateral wall in the A4C view showed the second-lowest quality. Across all views, particularly in the mid and apical segments, the outer (epicardial) border consistently scored lower than the endocardial border. These findings suggest that MRI should be considered when anterior-wall assessment is clinically essential—e.g., after myocardial infarction or in research settings requiring high-precision regional function data, for example, cardiotoxicity studies [3,10]. Notably, clear delineation of ≥5 borders was achieved in only 30% of studies, whereas ≥5 endocardial segments were adequately visualised in approximately 65% (Figure 3), underscoring the high prevalence of suboptimal image quality.

To overcome poor image quality in TTE, the implementation of standardised acquisition protocols remains of critical importance. Three-dimensional transoesophageal echocardiography (3D TEE), while not primarily designed for isolated assessment of the left ventricle, represents an excellent example of how protocol standardisation can lead to high image quality for multiple cardiac structures, particularly the cardiac valves. Previous studies have demonstrated that a standardised 3D TEE acquisition protocol significantly improves spatial resolution, border definition, and inter-observer reproducibility [3,8,9,10,31]. These principles are directly transferable to three-dimensional TTE, where careful optimisation of temporal and spatial resolution—and their mutual balance—is essential for achieving high-quality left ventricular imaging. Advantages of 3D echocardiography may be especially relevant in patients with implanted ICD/CRT-D, where device leads, pockets, and post-implant anatomical alterations may degrade acoustic windows and complicate border delineation [31,32]. High-quality imaging is essential not only for functional assessment but also for accurate risk stratification and longitudinal follow-up in CIED patients [32].

In our study, machine learning analysis identified no single predominant determinant of image quality. Instead, impaired visualisation correlated broadly with risk factors for heart failure (history of AF/MI, diabetes type 2, male), chamber dilation (LVEDV, wall segment hypokinesis), and patient-specific factors known to degrade acoustic windows (BMI, BSA). Beyond these factors, additional anatomical determinants should be considered [33,34]. In particular, pectus excavatum and concave chest wall conformations have been increasingly recognised as important contributors to poor acoustic windows, increased inter-rater variability, and reduced reproducibility of echocardiographic measurements [33,34]. Recent studies demonstrate that such chest wall deformities can affect ventricular volume and strain assessment, even in examinations otherwise deemed technically adequate, underscoring that chest wall anatomy represents a key determinant of echocardiographic image quality [33,34].

Overall, our scoring system delineates the key elements that warrant focused attention during image acquisition, offers realistic expectations of achievable image quality in heart failure populations, and clarifies the patient and cardiac characteristics most likely to influence visualisation. These insights may help experienced echocardiographers recognise recurrent imaging pitfalls and support trainees in developing strategies to obtain higher-quality images in routine practice consistently.

### Limitations and Future Directions

Our echocardiographic image-quality assessment was not validated against external reference standards such as cardiac MRI, which would allow more objective quantification of bias and variability in key metrics, including LVEF, beyond inter- and intra-observer reproducibility [2]. Incorporating MRI-derived parameters could strengthen the methodological framework and better define the impact of suboptimal imaging on quantitative analyses.

The cross-sectional design limits any inference regarding the prognostic relevance of image quality in relation to clinical outcomes [35,36,37]. It remains unclear whether prospective evaluation of TTE image quality, integrated with deformation imaging, could improve risk stratification beyond strain metrics alone. The potential application of an image-quality-based “correction factor” for strain measurements represents a promising concept for future randomised trials.

Furthermore, although the initial cohort included 913 patients, only 230 were available for the final analysis after applying the predefined inclusion criteria. This reduction raises the possibility of selection bias and may limit the generalizability of the results, mainly due to the absence of a TTE study (Figure 1). This may have introduced selection bias, favouring patients with complete imaging. This could influence the observed distribution of image quality across clinical subgroups [35,36,37]. A direct comparison between included and excluded patients could not be performed because exclusions were primarily due to missing or incomplete imaging and clinical data, precluding meaningful comparative analysis.

In addition, the primary aim of the study was the assessment of the quality of source imaging data rather than vendor-dependent quantitative parameters. All imaging data were acquired and analysed using General Electric (GE) platforms; therefore, the applicability of these findings to other vendors (e.g., Philips, Canon, and Siemens) remains uncertain, particularly with regard to derived parameters such as strain measurements. However, vendor-related differences are expected to have a limited impact on the quality of the raw echocardiographic cine loop. Moreover, the study population consisted exclusively of patients with heart failure, and extrapolation to non-heart failure populations should be undertaken with caution.

In this study, we deliberately focused on the development and evaluation of an echocardiographic image quality assessment framework rather than on linking image quality to downstream clinical or procedural endpoints such as response to cardiac resynchronisation therapy, device implantation feasibility, strain analysis, left ventricular quantification, or procedural planning metrics. Future studies are planned to formally evaluate the relationship between the proposed image quality score and specific clinical, functional, and procedural outcomes.

Future studies should aim to validate image-quality scoring systems against gold-standard modalities, such as MRI, and assess whether combining image-quality metrics with advanced echocardiographic parameters enhances prognostic accuracy. Expanding research to include patients with structural heart disease or post-infarction remodelling will clarify whether determinants of image quality identified in the HFrEF cohort also apply in other clinical contexts. Finally, the development of standardised, automated, guideline-directed, and potentially AI-assisted image-quality assessment tools may reduce subjectivity and strengthen clinical applicability in routine echocardiographic practice.

## 5. Conclusions

We introduce a reliable, fine-grained scoring framework for assessing TTE image quality in apical views. Using this system, we found substantial variation in segmental visualisation, with the anterior and anterolateral walls consistently demonstrating the poorest quality and inferior segments the best. Mid- and apical-level outer borders were systematically less well defined than endocardial borders. Overall, clear inner-edge-to-outer-edge delineation of at least five segmental borders was achieved in only one-third of studies. In contrast, at least five endocardial border segments were visualised in two-thirds of cases. No single factor uniformly predicted poor image quality. Overall, the prevalence of imaging challenges in TTE examinations performed in patients qualified for CRT-D/ICD implantation is high. These findings underscore the need for thorough training of echocardiographers and for sustained attention to technical details affecting image quality in order to achieve consistently high-quality images in routine practice.

## Figures and Tables

**Table 1:** Comparison of current approaches to echocardiographic image quality assessment and related methodologies.

Method/Approach	Description	Key Strengths	Limitations
TTE strain analysis (speckle tracking) [3,10,11]	Two/three-dimensional speckle tracking for myocardial deformation	Widely used clinically; quantified functional metrics	Moderate inter-vendor and inter-observer variability; limited reproducibility in poor image quality
Cardiac MRI as ground truth [12]	CMR feature tracking or tagging for deformation	High spatial resolution; excellent test–retest performance	Expensive, less accessible; not real-time
AI-derived quality metrics [15,16]	Deep learning models to assess or enhance image quality	Automated, can reduce observer bias and improve reproducibility	Dependent on training data and quality, ground truth labelling varies
Automated AI-based strain/LV function quantification [13,14]	DL methods for automated GLS/LVEF	Reduced test–retest variability; consistent measurements	Needs robust validation against clinical outcomes; Implementation in routine clinical workflow is still limited
Vendor-neutral platforms [17]	Software that standardises analysis across vendor systems	Reduces vendor variability; facilitates harmonised reporting	Still emerging; requires multi-centre validation
Proposed framework (fine-grained border assessment)	Structured manual assessment of endocardial and epicardial border delineation at the segmental level, combined with global qualitative image quality parameters	High anatomical granularity; explicit evaluation of inner and outer myocardial borders; vendor-independent; captures both local and global image quality determinants	Time-consuming; requires trained readers; currently manual, though well-suited for future automation using AI-based tools

**Table 2:** Demographic characteristics of patients included in the study.

Variable	All	CRT-D	ICD	*p*-Value
Count	230	114	116	-
Age	66 (64–67)	68 (65–70)	65 (61–65)	0.013
Males	182 (79)	89 (78)	93 (80)	0.818
Device implantation recommendation class: I	179 (78)	81 (71)	98 (85)	0.052
Device implantation recommendation class: IIa	51 (22)	33 (29)	18 (16)	
NYHA: II	165 (72)	76 (67)	89 (77)	0.122
NYHA: III	65 (28)	38 (33)	27 (23)	
HF decompensation in the last 12 months	53 (23)	23 (20)	30 (26)	0.386
History of AF/Aflutter	90 (39)	40 (35)	50 (43)	0.267
CAD	201 (87)	103 (90)	98 (85)	0.254
MI (STEMI/NSTEMI)	131 (57)	61 (54)	70 (60)	0.361
PCI/CABG	142 (62)	70 (61)	72 (62)	1.000
History of smoking	73 (32)	29 (25)	44 (38)	0.058
Arterial hypertention	220 (96)	110 (97)	110 (95)	0.768
COPD	46 (20)	17 (15)	29 (25)	0.081
Hypercholesterolemia	209 (91)	105 (92)	104 (90)	0.677
Diabetes type 2	88 (38)	39 (34)	49 (42)	0.264
BMI	28 (27–28)	27 (27–28)	28 (27–29)	0.276
BSA	2 (2–2)	2 (2–2)	2 (2–2)	0.050
eGFR (CKD-EPI)	67 (62–68)	66 (60–68)	69 (62–70)	0.313
eGFR (MDRD)	52 (51–54)	53 (50–55)	52 (50–55)	0.862
Sartans	25 (11)	13 (11)	12 (10)	0.963
Diuretics	171 (74)	90 (79)	81 (70)	0.152
Antiplatalet	43 (19)	18 (16)	25 (22)	0.341
MRA	184 (80)	97 (85)	87 (75)	0.081
ARNI	39 (17)	15 (13)	24 (21)	0.178
ACEi	135 (59)	68 (60)	67 (58)	0.875
Beta-blocker	216 (94)	106 (93)	110 (95)	0.757
ASA	111 (48)	59 (52)	52 (45)	0.358
NOAC	93 (40)	46 (40)	47 (41)	1.000
Vit. K antagonist	18 (8)	9 (8)	9 (8)	1.000
Statin	184 (80)	90 (79)	94 (81)	0.817
Digoxin	20 (9)	9 (8)	11 (10)	0.847
SGLT2i	55 (24)	24 (21)	31 (27)	0.393
Amiodarone	71 (31)	36 (32)	35 (30)	0.930

Continuous variables were expressed as medians with 95% confidence intervals. Categorical variables were summarised as counts and percentages.

**Table 3:** Electrocardiographic, echocardiographic, and image quality data of the analysed TTE studies.

Variable	All Studies *n* = 268	CRT-D Studies *n* = 135	ICD Studies *n* = 133	*p*-Value
Electrocardiographic data				
LBBB	123 (46)	113 (84)	10 (8)	<0.001
RBBB	22 (8)	19 (14)	3 (2)	<0.001
IVCD	29 (11)	5 (4)	24 (18)	<0.001
QRS duration [ms]	140 (135–143)	165 (163–169)	112 (109–114)	<0.001
Echocardiographic data				
LVEDV Bi-plane [mL]	199 (199–216)	208 (205–231)	189 (187–209)	0.049
LVESV Bi-plane [mL]	148 (146–161)	153 (152–175)	138 (135–153)	0.023
LVEF Bi-plane [%]	27 (26–27)	26 (25–27)	28 (26–28)	0.015
Moderate/severe TR	37 (14)	20 (15)	17 (13)	0.760
Moderate/severe MR	128 (48)	72 (53)	56 (42)	0.086
AF during the TTE study	16 (6)	7 (5)	9 (7)	0.773
Total image quality score for all views	119 (117–122)	120 (116–124)	118 (115–123)	0.685
Total border score for all views	78 (77–81)	78 (77–82)	79 (76–82)	0.985
Total first-sight image quality for all views	9 (8–9)	9 (8–9)	9 (8–9)	0.144

Continuous variables were expressed as medians with 95% confidence intervals. Categorical variables were summarised as counts and percentages.

**Table 4:** Internal reliability assessment of the image quality questionnaire.

Intra-Observer						
Variable	ICC	CV [%]	Mean Bias	Lower Limit of Agreement 95% CI	Upper Limit of Agreement 95% CI	Absolute Percentage Error [%]
Total border score	>0.9 *	19.9	0.7	−2.1	3.4	4.9
Total image quality score	>0.9 *	16.8	0.5	−2.8	3.9	4.0
Inter-observer						
Total border score	>0.9 *	19.0	0.8	−3.8	5.5	8.8
Total image quality score	>0.9 *	16.1	1.3	−4.1	6.8	7.9

ICC—intra-class correlation coefficient. * *p* < 0.001.

## Data Availability

The data underlying this article will be shared on reasonable request to the corresponding author (WN). Reason: privacy and ethical restrictions.

## Supplementary Materials

The following supporting information can be downloaded at: https://www.mdpi.com/article/10.3390/jcm15031055/s1.
