# Supplementary material for: Development of a Framework for Echocardiographic Image Quality Assessment and Its Application in CRT-D/ICD Patients
Source: J Clin Med. 2026 Jan 28;15(3):1055. doi: 10.3390/jcm15031055 (PMC12898213; doi:10.3390/jcm15031055)
Supplement: Supplementary file 1 [file jcm-15-01055-s001.zip › jcm-4106492-supplementary/Supplementary material S1.pdf]

Supplementary material S1

Results

Echocardiogram image quality analysis in ICD patients

In patients qualified for ICD implantation, overall image quality and the total number of border points increased significantly across apical projections. The median total overall image quality score was 37.0 (95% CI: 36.2–39.2) in the a2c view, 40.0 (95% CI: 37.6–40.6) in the a3c view, and 43.0 (95% CI: 41.3–44.0) in the a4c view ($p<0.001$).

Similarly, the median total border points increased from 25.0 (95% CI: 24.0–26.3) in a2c to 26.0 (95% CI: 24.9–27.2) in a3c, and 28.0 (95% CI: 27.5–29.4) in a4c ($p<0.001$).

The image axis alignment was correct in 74% of a2c views, compared with approximately 90% in both a3c and a4c views ($p<0.001$). The apex was adequately visualized in 89% of a2c views, 92% of a3c views, and 99% of a4c views ($p=0.006$).

Numerous artefacts were most frequently observed in a2c projection, affecting 24% of examinations, compared with 19% in a3c and 13% in a4c views ($p=0.043$). Similarly, gain adjustment was most often suboptimal in a2c views (24%), compared with 19% in a3c and 15% in a4c views ($p=0.171$).

Regarding border delineation, five or more well-visible borders were identified in 25% of a2c, 32% of a3c, and 35% of a4c studies ($p=0.059$). For well-visible endocardium, five or more points were scored in 55%, 63%, and 71% of a2c, a3c, and a4c studies, respectively ($p=0.341$).

Echocardiogram image quality analysis in CRT-D patients

In patients qualified for CRT-D implantation, the total overall image quality score and total border points differed significantly across apical projections. The median total overall image quality score increased progressively from 38.0 (95% CI: 36.5–39.3) in the a2c view to 40.0 (95% CI: 38.5–41.6) in the a3c view, and 43.0 (95% CI: 41.2–44.0) in the a4c view ($p<0.001$).

Similarly, the median total border points were 24.0 (95% CI: 23.8–25.8) for a2c, 26.0 (95% CI: 25.6–27.9) for a3c, and 28.0 (95% CI: 27.2–29.2) for a4c ($p<0.001$).

Correct alignment of the imaging axis was achieved in 77% of a2c acquisitions, whereas the a3c and a4c views demonstrated higher rates of adequate alignment, at 86% and 96%, respectively ($p<0.001$). The apex was clearly depicted in 82% of a2c recordings, improving to 89% in a3c and 96% in a4c images ($p=0.001$).

Artefacts occurred most frequently in the a3c view, where they were noted in 22% of studies, compared with 21% in the a2c and 17% in the a4c view ($p=0.043$). Suboptimal gain settings were most common in the a2c view (22%), with slightly lower rates in the a3c (20%) and a4c (18%) views ($p=0.415$).

Assessment of border visibility showed that at least five clearly defined borders were present in 18% of a2c studies, compared with 32% in a3c and 36% in a4c examinations ($p=0.025$). Similarly, scoring of endocardial visibility indicated that five or more points were achieved in 57% of a2c, 65% of a3c, and 68% of a4c studies ($p=0.823$).
